# An Audit of Adherence to the Pre-Referral Process for Acute Inpatient Admissions in a Male and a Female Acute Inpatient Unit Over Six Months in Birmingham and Solihull Mental Health Foundation Trust (BSMHFT)

**DOI:** 10.1192/bjo.2022.458

**Published:** 2022-06-20

**Authors:** Radhika Lakhani, Selvaraj Vincent, Isaac Nyemitei-Addo, Kishan Patel-Smith, Priyavarshini Ramesh

**Affiliations:** 1 South West London and St George's NHS Foundation Trust, London, United Kingdom; 2 Birmingham and Solhull Mental Health Foundation Trust, Birmingham, United Kingdom; 3 Birmingham Medical School, Birmingham, United Kingdom

## Abstract

**Aims:**

In view of the limited number of acute inpatient beds relative to demand in England, a thorough assessment prior to referral is paramount in ascertaining clinical need. A comprehensive risk assessment is crucial in light of patient safety and assessing risk to others. Moreover, the appropriateness of an acute bed should be considered, and whether psychiatric intensive care or forensic services may be more appropriate for the patient. In line with this, the Birmingham and Solihull Mental Health Foundation Trust (BSMHFT) admissions policy details standards of the assessment prior to referral to acute inpatient services. Pre-referral assessment should be carried out by a multidisciplinary team including a senior doctor. It should include rationale and plan of care for admission, risk assessment and section status on admission alongside type of bed being requested. Referrals are accepted from multiple teams including Home Treatment, the Place of Safety and Liaison Psychiatry. Aim: To audit adherence to the pre-referral policy for acute inpatient admissions to a male and female ward in BSMHFT, including comprehensive assessment, plan of care and consideration of appropriate bed type.

**Methods:**

A retrospective audit of pre-referral documentation for all admissions from April to September 2019 to a male and separate female acute inpatient unit at the Zinnia Centre, Birmingham was carried out. This included 83 male admissions and 82 female admissions. Documentation was reviewed on the clinical system Rio. Parameters reviewed included assessing clinician, assessment summary, capacity assessment, consideration of bed type, plan of care and section details.

**Results:**

Overall, almost half of admissions (49%) were assessed by a full Mental Health Act team, 34% by a senior psychiatric doctor and the remainder by psychiatric nurses in the referring department. An up-to-date assessment summary was completed in the majority of cases (67%) prior to referral. Risk assessments were completed in 82% of cases. 35% of cases included a detailed plan of care which met audit standards. Capacity assessment alongside outcome was documented in 13% of cases. The type of bed was only considered in 13% of cases.

**Conclusion:**

Whilst assessment and risk documentation was completed in the majority of cases, few cases had a clear plan of care and appropriateness of bed type was rarely considered in assessment. Greater adherence to the pre-referral process could facilitate treatment decisions during admission and seek to ensure a safer inpatient environment.

